# Complete Genome Sequence of Porcine Encephalomyocarditis Virus Strain BD2

**DOI:** 10.1128/genomeA.01028-13

**Published:** 2013-12-12

**Authors:** Wanzhe Yuan, Xiuyuan Zhang, Jiguo Sun

**Affiliations:** College of Animal Medicine, Agricultural University of Hebei, Baoding, Hebei, Chinaa; Hebei Engineering and Technology Research Center of Veterinary Biological Products, Baoding, Hebei, Chinab; North China Research Center of Animal Epidemic Pathogen Biology, China Agriculture Ministry, Baoding, Hebei, Chinac

## Abstract

Encephalomyocarditis virus (EMCV) causes acute myocarditis in young pigs or reproductive failure in sows, and it is divided into two main groups. Here, we report the complete genome sequence of EMCV strain BD2, which belongs to group I.

## GENOME ANNOUNCEMENT

Encephalomyocarditis virus (EMCV) has been recognized worldwide as a pathogen infecting many species, including pigs, rodents, cattle, elephants, raccoons, marsupials, and primates, such as baboons, monkey, chimpanzees, and even humans (1–4). The virus causes acute myocarditis in young pigs or reproductive failure in sows, and it has been reported in many areas with swine industries, including Europe and Asia. Using Chinese serological and etiological methods, it was confirmed that EMCV infection occurred in many pig farms in recent years (5).

EMCV belongs to the *Cardiovirus* genus of the *Picornaviridae* family. Its genome is a single-stranded positive-sense RNA with a size of approximately 7.8 kb nucleotides, and it contains a large open reading frame (ORF) (6). EMCV strain BD2 was isolated from tissue samples from pigs with anorexia, rapid breathing, and acute myocarditis in a commercial pig farm in Hebei province, China, in 2009. The full-length genome of the isolated strain was amplified using EMCV-specific primers. The PCR products were purified and cloned into the pEASY-Blunt cloning vector (TransGen, China) and subsequently were subjected to an automated sequencing reaction (Invitrogen, China). The terminal sequences were acquired by using a kit for rapid amplification of cDNA ends (TaKaRa, Japan). Genomic analyses were conducted using DNAman (University of California). Phylogenetic trees were constructed using MEGA5. The complete genomic sequence of BD2 was determined to be 7,741 nucleotides in length, with a G+C content of 48.71%. The ORF of BD2 encodes 11 proteins that are similar to those reported previously for other EMCV strains, i.e., VP1 to VP4, 2A to 2C, and 3A to 3D, as well as a virus-leading protein (L protein) (7, 8). Multiple sequence alignment based on the ORF was completed by means of the DNAStar software (DNAStar, Madison, WI), using other available strains from the GenBank nucleotide database. BD2 shares a high identity (98.3 to 100%) with EMCV strains NJ08, BEL-2887A/91, CBNU, K3, K11, GX0601, GXLC, pEC9, BJC3, HB1, and PV21, and it shares a lower sequence identity (73.7 to 90.7%) with strain PV2 and the EMCV-B and EMCV-D variants. The phylogenetic tree based on the ORF nucleotide sequences showed that all EMCV isolates belonged to two main groups (group I and II). BD2 strain belongs to group I with the other Chinese EMCV strains. The relationship between the genomic information and pathogenicity needs to be further investigated.

### Nucleotide sequence accession number.

The genome sequence of EMCV strain BD2 has been deposited in GenBank under the accession no. KF709977.1.
